# The Loss of Temporal Muscle Volume is Associated with Poor Outcome in Patients with Subarachnoid Hemorrhage: An Observational Cohort Study

**DOI:** 10.1007/s12028-023-01751-z

**Published:** 2023-06-12

**Authors:** Mario Kofler, Philipp Reitmeir, Bernhard Glodny, Verena Rass, Anna Lindner, Bogdan A. Ianosi, Max Gaasch, Alois J. Schiefecker, Lauma Putnina, Ronny Beer, Paul Rhomberg, Erich Schmutzhard, Bettina Pfausler, Raimund Helbok

**Affiliations:** 1 Neurological Intensive Care Unit, Department of Neurology, Medical University of Innsbruck, Innsbruck, Austria; 2 Department of Radiology, Medical University of Innsbruck, Innsbruck, Austria; 3 University for Health Sciences, Medical Informatics and Technology, Hall in Tirol, Austria; 4 Department of Neuroradiology, Medical University of Innsbruck, Innsbruck, Austria; 5 Department of Neurology, Johannes Kepler University Linz, Linz, Austria; 6 Department of Anaesthesiology and Intensive Care Medicine, Medical University of Innsbruck, Innsbruck, Austria

**Keywords:** Subarachnoid hemorrhage, Muscle wasting, Intensive care, Neurocritical care, Biomarker

## Abstract

**Background:**

Intensive care unit (ICU) acquired weakness is a major contributor to poor functional outcome of ICU patients. Quantification of temporal muscle volume assessed on routine computed tomography (CT) scans may serve as a biomarker for muscle wasting in patients suffering from acute brain injury.

**Methods:**

This is a retrospective analysis of prospectively collected data. Temporal muscle volume was assessed on head CT scans of consecutive patients with spontaneous subarachnoid hemorrhage within prespecified time frames (on admission, then weekly ± 2 days). Whenever possible, temporal muscle volume was assessed bilaterally and averaged for the analysis. Poor functional outcome was defined as a 3-month modified Rankin Scale Score ≥ 3. Statistical analysis was performed using generalized estimating equations to handle repeated measurements within individuals.

**Results:**

The analysis comprised 110 patients with a median Hunt & Hess score of 4 (interquartile range 3–5). Median age was 61 (50–70) years, 73 patients (66%) were women. Baseline temporal muscle volume was 18.5 ± 0.78 cm^3^ and significantly decreased over time (*p* < 0.001) by a mean of 7.9% per week. Higher disease severity (*p* = 0.002), hydrocephalus (*p* = 0.020), pneumonia (*p* = 0.032), and bloodstream infection (*p* = 0.015) were associated with more pronounced muscle volume loss. Patients with poor functional outcome had smaller muscle volumes 2 and 3 weeks after subarachnoid hemorrhage compared with those with good outcome (*p* = 0.025). The maximum muscle volume loss during ICU stay was greater in patients with poor functional outcome (− 32.2% ± 2.5% vs. − 22.7% ± 2.5%, *p* = 0.008). The hazard ratio for poor functional outcome was 1.027 (95% confidence interval 1.003–1.051) per percent of maximum muscle volume loss.

**Conclusions:**

Temporal muscle volume, which is easily assessable on routine head CT scans, progressively decreases during the ICU stay after spontaneous subarachnoid hemorrhage. Because of its association with disease severity and functional outcome, it may serve as a biomarker for muscle wasting and outcome prognostication.

## Introduction

Despite advances in the critical care management, spontaneous subarachnoid hemorrhage (SAH) remains a devastating disease and often leads to severe morbidity in survivors [1]. Besides factors associated with primary and secondary brain injury, intensive care unit (ICU) acquired weakness (ICUAW) largely impacts functional outcome, especially in patients with severe disease and prolonged ICU stay [2, 3].

ICUAW comprises muscle volume loss through protein catabolism and disuse [3, 4], as well as functional impairment, structural changes, and atrophy by the occurrence of critical illness polyneuropathy (CIP) and critical illness myopathy (CIM) [2, 4]. These mechanisms commence within hours of critical illness onset and lead to weakness of the extremities and respiratory muscles [3]. ICUAW has been associated with prolonged mechanical ventilation, longer ICU stay, and increased mortality [3–5]. Even after ICU discharge, a substantial number of patients suffer from long-lasting sequelae and impaired quality of life [2, 6, 7].

The clinical diagnosis of ICUAW is challenging in sedated patients and those developing delirium, encephalopathy, and focal neurological deficits, which are common conditions in the neurocritical care unit. Besides electrophysiological investigations (electroneurography, electromyography, direct muscle stimulation) [2] and biopsies to objectify muscular and nerval dysfunction (or structural damage, respectively) [2, 8], imaging techniques have been used to quantify muscle wasting.

Repeated muscular ultrasound measurements demonstrated a significant decrease during the early phase of critical illness, which was more pronounced in patients suffering from multiorgan failure [8]. However, the methodological variability of ultrasound studies has led to a lack of consistent and reproducible protocols and therefore unclear validity of this operator-dependent technique [9]. Obesity and tissue edema, which is common in people who are critically ill, also constitute limitations to sonographic investigations [2].

Computed tomography (CT) and magnetic resonance imaging have also been used to assess muscle volume loss in critically ill patients, focusing on the lower extremities [10, 11]. Although these modalities would overcome most of the limitations of ultrasound measurements, their routine clinical use for the quantification of muscle wasting is hindered by ionizing radiation (through CT) and the accruing necessity of patient transport to the radiology department.

The temporal muscle arises from the temporal fossa and the temporal fascia and is therefore depicted on routine head CT scans. Although cranial nerves appear to be less affected by CIP [12], it is conceivable that the temporal muscle may undergo similar critical illness-induced changes as other skeletal muscles. In line with this, a correlation between temporal muscle thickness and lumbar skeletal muscle cross-sectional area (a marker of sarcopenia) was found in patients with metastatic cancer [13].

In this study, we intended to measure temporal muscle volume on repeated head CT scans obtained as part of clinical routine in patients with SAH. We hypothesized that temporal muscle volume would decrease over the course of the ICU stay and that a higher degree of muscle volume loss is associated with adverse functional outcome.

## Methods

### Study Design and Patient Population

This is a retrospective analysis of prospectively collected data from 255 screened consecutive patients with nontraumatic SAH admitted to the neurocritical care unit of a tertiary referral center between April 2010 and November 2016. The study was approved by the ethics committee of the Medical University of Innsbruck (AN3898 285/4.8). Informed consent was obtained from all patients according to federal regulations. Inclusion criteria were the diagnosis of nontraumatic SAH, patient age above 18 years, and the availability of noncontrast enhanced head CT scans performed on admission and at least one performed 5–9 days or 12–16 days after admission. Patients were excluded if they were admitted to the neurocritical care unit 2 or more days after the hemorrhage, if they were transferred to another unit within 7 days of admission, and if the available CT scans were of insufficient quality for the measurement of temporal muscle volume.

### Patient Treatment

Patients were treated according to current international guidelines for the management of SAH [14, 15], with the exception of nimodipine being administered intravenously in poor-grade patients. Angiographically detected ruptured aneurysms were treated early by either neurosurgical clipping or endovascular coiling after interdisciplinary discussion. If no aneurysm was detected, digital subtraction angiography was repeated approximately 2 weeks after the hemorrhage. An external ventricular drain was placed in case of hydrocephalus. Transcranial color-coded duplex sonography was used for vasospasm screening. If severe vasospasm was detected, confirmatory conventional angiography and the administration of intraarterial nimodipine were considered. Delayed cerebral ischemia was defined as either a new focal neurological deficit, a decrease of two or more points on the Glasgow Coma Scale, or the detection of a new infarct on cerebral CT or magnetic resonance imaging scans, not attributable to other causes [16]. In the presence of severe vasospasm or delayed cerebral ischemia, arterial blood pressure was augmented and normothermia and a CO_2_ partial pressure of 40–45 mm Hg were targeted. Mechanical ventilation and sedation were performed as clinically indicated.

### Grading, Definitions, and Follow-Up

Clinical disease severity was graded using the Hunt & Hess (H&H) scale on admission [17]. H&H grades 1–3 were considered as good grade and H&H grades 4–5 were considered poor grade. An independent neuroradiologist rated all CT scans on admission using the modified Fisher scale [18]. Infectious complications were diagnosed by using the Centers for Disease Control and Prevention criteria. A trained study nurse, blinded to clinical and radiographic data, assessed functional outcome 3 months after the hemorrhage using the modified Rankin Scale. In three patients, the 3-month outcome was missing and was carried forward from discharge. A modified Rankin Scale score of 0–2 was defined as favorable outcome and a score of 3–6 was defined a as poor functional outcome.

### Temporal Muscle Volume Measurement

Because we were interested in temporal dynamics of muscle volume, we only included CT scans within certain time frames to improve comparability: admission; 5–9 days after admission (1 week); 12–16 days after admission (2 weeks); 19–23 days after admission (3 weeks); 26–30 days after admission (4 weeks); 33–37 days after admission (5 weeks); and 40–44 days (6 weeks). For the assessment of temporal dynamics, not only were absolute muscle volumes compared, but relative differences (expressed as percentages) in muscle volume between the predefined intervals and the baseline values were calculated.

The maximum muscle volume decrease refers to the relative difference between baseline volume and the lowest muscle volume measured during the ICU stay. All available CT scans within the predefined periods were assessed for sufficient quality. Only one CT scan per patient per timeframe was analyzed. Whenever possible, temporal muscle volume was assessed bilaterally and averaged for the analysis. In case of cranial surgery, only the unaffected hemisphere was measured. Scans were excluded if neither side was assessable due to artifacts.

**Fig. 1:**
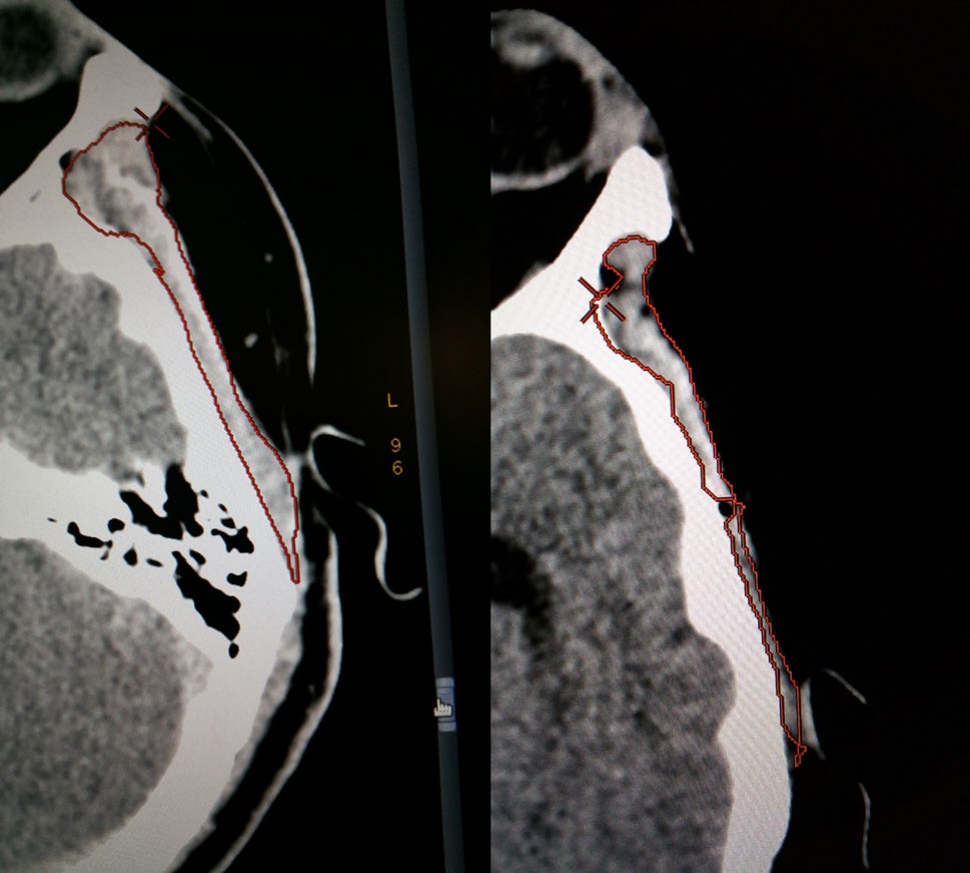
Manually marked and afterward 3D-reconstructed measurement of muscle volume using the AW volume share 5 software (GE Healthcare, Chicago, IL)

A single rater, blinded to clinical data, performed all measurements using anonymized images in random order to avoid bias. Noncontrast enhanced CT scans with a slice thickness of 4 mm were used. Muscle volume was measured between the zygomatic arch (first slice depicting the completely closed arch) and the inferior temporal line. These landmarks were chosen because the temporal muscle is located very distinctively between the cranial bones (inside) and the temporal fascia (outside) within this area. The muscle was manually marked and 3D-reconstructed afterward using the AW volume share 5 software (GE Healthcare, Chicago, IL) (Fig. 1).

An exploratory analysis revealed that 322 (89%) of all analyzed CT scans were performed within the first 3 weeks of ICU stay. In view of this distribution, only the CT scans performed during the first 3 weeks of ICU stay were included in the analysis regarding associations between temporal dynamics of muscle volume, disease severity, complications, and functional outcome.

### Data Collection, Presentation, and Statistical Analysis

All data were collected in our institutional SAH database, an ongoing, prospective single-center observational cohort including all patients with nontraumatic SAH. Interventions and complications were confirmed in weekly meetings of the study team and the treating neurointensivists. Categorical variables are presented as count and percentage, continuous variables as mean and standard error of mean or median and interquartile range. Comparisons between patient characteristics and baseline temporal muscle volume were performed using the Wilcoxon rank-sum test. Associations between maximum muscle volume loss and functional outcome were assessed in a generalized linear model and adjusted for relevant covariates as specified in the results. Analyses regarding repeated measurements (muscle volume over time) were performed utilizing generalized estimating equations choosing the matrix best fitting the data. Cases with missing values were included. All analyses were performed with IBM SPSS (IBM SPSS Statistics, Version 24.0. Armonk, NY). A *p* value < 0.05 was considered as statistically significant.

## Results

Of 255 screened consecutive patients with nontraumatic SAH, 98 didn’t meet inclusion criteria, 33 were excluded due to insufficient quality of cerebral CT scans, 12 were excluded due to early transfer to another ward, and 2 were excluded due to late admission, leaving 110 patients for final analysis.

Demographic data, admission details, interventions, complications, and outcomes of 110 consecutive patients with SAH fulfilling the inclusion criteria are shown in Table 1.

**Table 1:** Patient characteristics, grading, complications, and outcome

Patient characteristics (*n* = 110)	*n* (%) or median (IQR)
Age (year)	61 (50–70)
Female sex	73 (66)
Medical history of hypertension	51 (46)
Medical history of diabetes	6 (6)
Medical history of hypercholesterolemia	18 (16)
Medical history of smoking	41 (37)
Hunt & Hess grade on admission	
1	12 (11)
2	9 (8)
3	26 (24)
4	13 (12)
5	50 (45)
Loss of consciousness at ictus	62 (56)
Modified Fisher score (first CT scan)	
1	5 (4.5)
2	11 (10)
3	23 (21)
4	71 (65.5)
Aneurysm treatment	
Coiling	57 (52)
Clipping	45 (41)
None detected	6 (5)
No treatment (withhold of therapy)	2 (2)
Hydrocephalus (requiring EVD)	93 (85)
Delayed cerebral ischemia	31 (28)
Pneumonia	74 (67)
Urinary tract infection	31 (28)
Bloodstream infection	29 (26)
Duration of intubation (d)	14 (10–21)
Length of ICU stay (d)	29 (22–44)
Modified Rankin Scale score after 3 months	
0	1 (1)
1	19 (17)
2	18 (16)
3	12 (11)
4	16 (15)
5	24 (22)
6	20 (18)

*CT* computed tomography, *EVD* external ventricular drain, *ICU* intensive care unit, *IQR* interquartile range

Overall, 361 CT scans (3 [interquartile range 3–4] per patient) were analyzed. Baseline temporal muscle volume was 18.5 ± 0.78 cm^3^. Older age (*p* = 0.002) and female sex (*p* < 0.001) were associated with lower baseline muscle volume, whereas a medical history of hypertension (*p* = 0.286), diabetes mellitus (*p* = 0.151), hypercholesterolemia (*p* = 0.878), or smoking (*p* = 0.146) were not.

### Temporal Dynamics of Muscle Volume

**Fig. 2:**
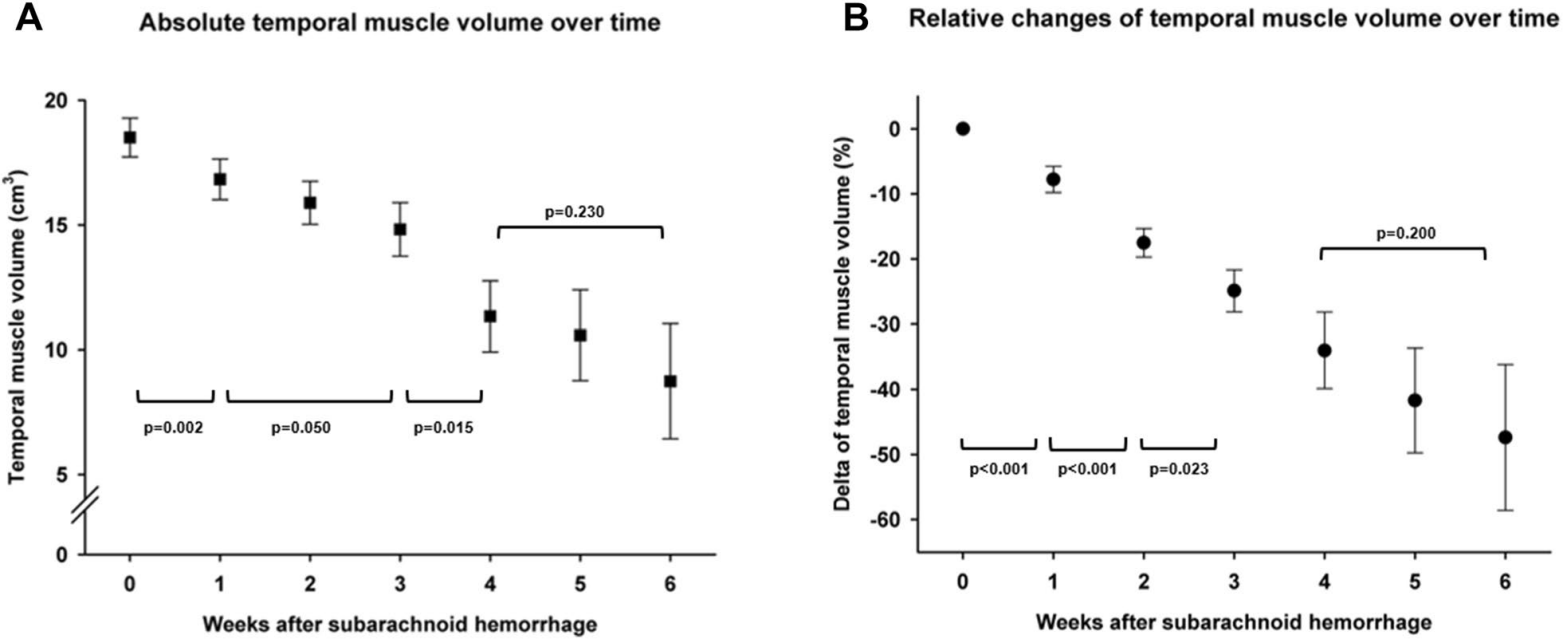
Temporal dynamics of absolute volume (**a**) and relative volume changes (**b**) of the temporal muscle of subarachnoid hemorrhage patients from admission to up to 6 weeks of ICU stay. Downward-facing brackets indicate significant changes over time, and upward-facing brackets nonsignificant changes over time. ICU, intensive care unit

Temporal muscle volume was largest at baseline and significantly decreased over time (*p* < 0.001, Fig. 2a). Accordingly, the relative reduction over time expressed in percentage change from baseline was highly significant (*p* < 0.001, Fig. 2b). The mean reduction of muscle volume was − 1.54 cm^3^/week and − 7.9% per week, respectively. The mean maximum muscle volume decrease of patients was − 27.9% ± 1.8%.

### Muscle Volume Loss and Disease Severity

**Fig. 3:**
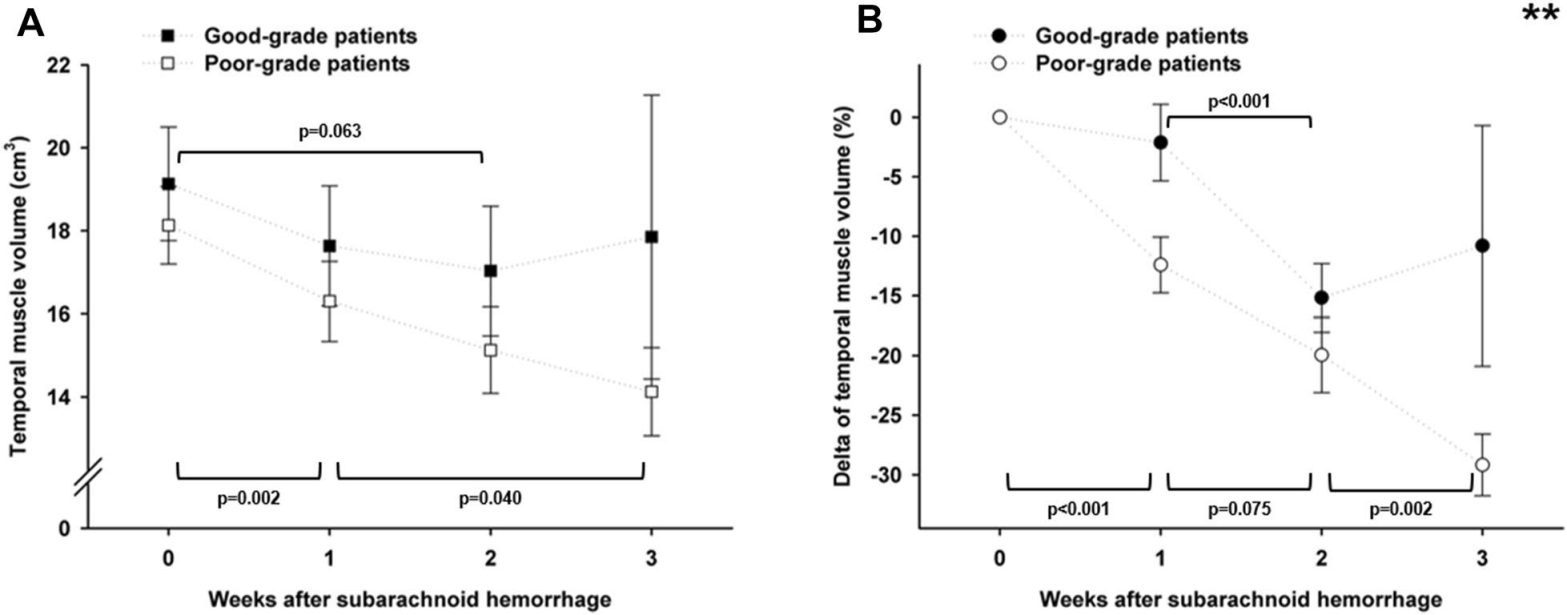
Temporal dynamics of absolute volume (**a**) and relative volume changes (**b**) of the temporal muscle from admission to 3 weeks of ICU stay in patients with good-grade (black squares and dots) and patients with poor-grade (white squares and dots) subarachnoid hemorrhage. Upward-facing brackets indicate the significance level of changes over time in good-grade patients, and downward-facing brackets indicate that in poor-grade patients. The asterisks (**) in plot B indicate an overall significant difference between good-grade and poor-grade patients (*p* < 0.01)

Baseline muscle volume did not differ based on admission H&H grade (*p* = 0.091, Fig. 2a). Within the first 3 weeks, poor-grade patients exhibited a progressive decrease of muscle volume of − 1.8 cm^3^/week and − 9.7% per week (Fig. 3a and 3b). In good-grade patients, there was no significant decrease of absolute muscle volume (Fig. 3a) and the percentage muscle loss was less pronounced compared with poor-grade patients (*p* = 0.002, adjusted for age and sex) with no dynamic after week 2 (Fig. 3b). The mean maximum muscle volume loss was greater in poor-grade patients compared with good-grade patients (− 33.1% ± 2.3% vs. − 20.8% ± 2.6%, *p* = 0.0081, adjusted for age, sex, and length of ICU stay).

### Aneurysm Treatment and Complications

Associations between temporal muscle volume loss, modality of aneurysm treatment, and hospital complications are shown in Table 2. The presence of hydrocephalus, pneumonia, and bloodstream infection were associated with greater muscle volume decrease, whereas no difference between the modality of aneurysm securing was observed.

**Table 2:** Aneurysm treatment, hemorrhage characteristics, complications, and temporal muscle volume loss

Variable	Absolute muscle volume loss (cm^3^)			Percentage muscle volume loss			Maximum muscle volume loss (%)		
	HR	CI 95%	*p* value	HR	CI 95%	*p* value	Yes, mean (± SEM)	No, mean (± SEM)	*p* value
Clipping (vs. coiling)	1.39	0.53–3.67	0.504	1.02	0.99–1.04	0.194	− 29.1 (± 1.8)	− 25.9 (± 2.7)	0.406
High modified Fisher grade^a^	1.05	0.34–3.23	0.932	1.00	0.94–1.06	0.971	− 28.3 (± 2.0)	− 25.5 (± 3.7)	0.593
Parenchymal hemorrhage	1.15	0.33–3.94	0.828	1.01	0.95–1.07	0.828	− 30.5 (± 3.5)	− 26.6 (± 2.1)	0.305
Hydrocephalus	4.69	0.94–23.4	0.060	1.15	1.02–1.29	0.020*	− 29.8 (± 1.9)	− 16.3 (± 4.0)	**0.007**
Delayed cerebral ischemia	1.72	0.57–5.24	0.340	1.01	0.96–1.07	0.714	− 28.4 (± 3.6)	− 27.6 (± 2.1)	0.839
Pneumonia	2.10	0.70–6.32	0.185	1.03	1.01–1.05	0.032*	− 30.4 (± 2.3)	− 22.7 (± 2.6)	**0.042**
Bloodstream infection	3.46	1.13–10.6	0.030*	1.10	1.02–1.19	0.015*	− 36.5 (± 3.6)	− 24.5 (± 2.0)	**0.002**
Urinary tract infection	2.12	0.42–10.6	0.362	1.04	0.96–1.12	0.360	− 29.4 (± 2.2)	− 23.5 (± 3.3)	0.146

*CI* confidence interval, *HR* hazard ratio, *SEM* standard error of mean

^a^Modified Fisher grade 3 or 4 (vs. 1 or 2)

*Bold *p*-values are <0.05

### Muscle Volume Loss and Functional Outcome

**Fig. 4:**
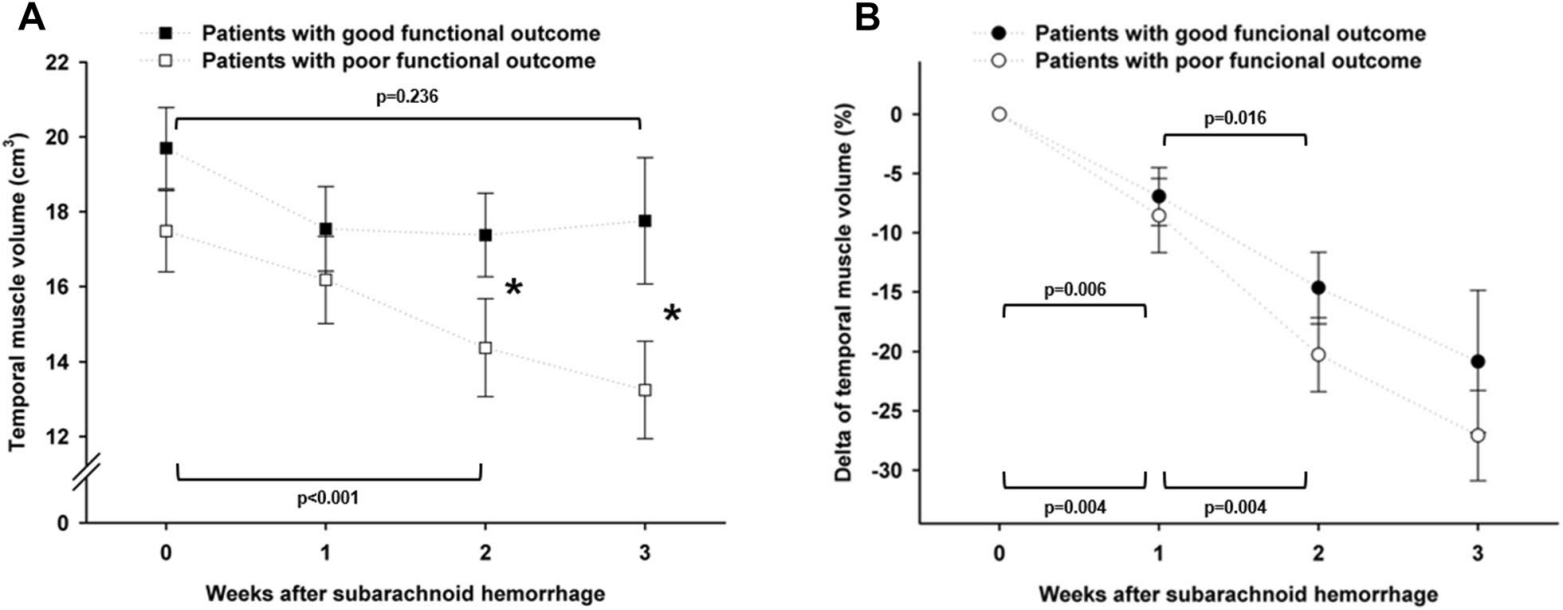
Temporal dynamics of absolute volume (**a**) and relative volume changes (**b**) of the temporal muscle from admission to 3 weeks of ICU stay in SAH patients with good (black squares and dots) and poor (white squares and dots) outcome after 3 months. Upward-facing brackets indicate the significance level of changes over time in patients with good, and downward-facing brackets indicate that in patients with poor outcome. The asterisks (*) in plot A indicate a significant difference in absolute muscle volumes 2 and 3 weeks after the ictus between patients with good or poor outcome (*p* < 0.05)

Patients with poor 3-month functional outcome had smaller absolute muscle volumes 2 and 3 weeks after SAH, compared with those with good outcome (*p* = 0.025), and exhibited a significant decrease of absolute muscle volume over time, which was not observed in patients with good outcome (Fig. 4a). A relative loss of muscle volume was present in both groups with poor-grade patients showing a trend toward a more pronounced decrease (*p* = 0.093, Fig. 4b). The maximum muscle volume loss was greater in patients with poor functional outcome (− 32.2% ± 2.5% vs. − 22.7% ± 2.5%, *p* = 0.021). The hazard ratio for poor functional outcome was 1.027 (95% confidence interval 1.003–1.051) per percent of maximum muscle volume loss. The analyses were adjusted for age, sex, H&H grade, and length of ICU stay.

## Discussion

In this study, we describe a significant and progressive loss of temporal muscle volume in patients with subarachnoid hemorrhage over the course of ICU stay. Disease severity and hospital complications, including hydrocephalus, pneumonia, and bloodstream infection, were associated with a more pronounced decrease. Moreover, the extent of temporal muscle loss was associated with functional outcome after 3 months.

Here, we propose a novel and simple method of measuring skeletal muscle loss of ICU patients using routine head CT scanning. So far, imaging studies have focused on quantifying muscle wasting of the lower extremities [9–11]. Serial ultrasound measurements of the rectus femoris cross-sectional area (CSA) revealed a muscle volume loss of about 2% per day [8]. Similarly, repeated CT assessment of the quadriceps muscle volume showed a decrease of muscle mass of 2.9% per day [10]. The extent of temporal muscle volume decrease in the current study was lower (7.9% per week), which may be explained by the higher impact of CIP/CIM on peripheral nerves and muscles [12]. It is not clear to what extent changes in temporal muscle volume are indicative of changes in other skeletal muscles. In recent studies comparing the psoas CSA (in patients with trauma) [19] and lumbar skeletal muscle CSA (in patients with metastatic cancer) [13] with temporal muscle thickness, the authors found a strong correlation. This supports our hypothesis that the proposed method of CT-based quantification of temporal muscle volume can be used as biomarker for overall muscle loss in patients with head injury. Moreover, we found an almost linear decrease of muscle volume over weeks, particularly in poor-grade patients. This is comparable to the previously described decline of lower extremity muscle mass in ICU patients [8].

The main advantage of measuring the temporal muscle volume (in contrast to sectional imaging of the lower extremities) is that it is depicted and easily identifiable on routine head CT scans, which are frequently performed in neurocritical care patients. Therefore, there is no need for additional patient transport, which is not only time-consuming but also potentially hazardous. Compared with muscular ultrasound, which is highly operator-dependent and lacking consistent methodology [9], the measurement technique is easily reproducible and may be less susceptible to interrater variability.

Our data indicate that a higher grade of disease severity is associated with a more pronounced decrease of muscle volume. On the one hand, marked disease severity itself may contribute to muscle wasting by promoting catabolism and proinflammatory cascades. Critical illness causes a diversion of nutrients away from the muscle and a release of muscle-bound amino acids into the circulation—a mechanism that is hypothesized to be an adaptive response to the increased metabolic demands of the body [20]. The result is a sharply increased rate of muscle protein breakdown and simultaneously decreased rate of protein synthesis. It was shown earlier that patients suffering from multiorgan failure experience a more drastic reduction of muscle mass than patients with single organ failure [8]. Inflammation, represented by increased interleukin-6 levels, was also shown to be one of the most important risk factors for the development of CIM [21]. Furthermore, inflammation-induced microcirculatory changes have been discussed as a pathophysiologic mechanism of axonal damage in CIP [22]. Sepsis (and accompanying systemic inflammation) therefore appears to be inducing both muscle volume loss and neuromuscular dysfunction [23]. In line with this, pneumonia and especially bloodstream infections were associated with a more pronounced loss of temporal muscle volume in our patients. Furthermore, a higher grade of disease severity comes along with a reduced level of consciousness in patients with SAH, which in turn promotes muscle disuse and prolonged immobility.

On the other hand, poor-grade patients often require intensified treatment. Deep sedation is a common treatment strategy in neurocritical care patients for the prevention and treatment of raised intracranial pressure. Although sedation interruption and early mobilization are strongly advised in various subgroups of intensive care patients, it may cause secondary brain injury in patients with acute brain injury [24]. This practice, however, interferes with the prevention of muscle wasting and may aggravate ICUAW by prolonging immobility [2]. Early passive mobilization, however, was regularly performed in our patients. Furthermore, commonly required substances including corticosteroids and muscle relaxants have been associated with ICUAW [25, 26], even though these findings could not be reproduced in other cohorts [2].

Hydrocephalus itself may be associated with ICUAW, as muscle weakness, especially of the lower extremities, was described in patients suffering from normal pressure hydrocephalus [27]. An association with muscle wasting has not been shown. Acute hydrocephalus and concomitant raised intracranial pressure appear to be associated with higher systemic levels of catecholamines [28]. In patients with pheochromocytoma, excess levels of catecholamines were associated with reduced skeletal muscle mass [29]. Furthermore, there is a well-established association between acquired hydrocephalus and inflammation, which may contribute to muscle volume loss [30]. The placement of an external ventricular drain, a surgical procedure, may also contribute to ICUAW by inducing proinflammatory cascades and the need for patient sedation.

The relative reduction of muscle volume appears to be a more reliable parameter than absolute decrease over time. This is due to the considerable interindividual variability of baseline temporal muscle volume, strongly influenced by age and sex, which has been shown earlier [31]. With respect to this, we adjusted our statistical models accordingly. Preexisting vascular risk factors were not associated with baseline muscle volume.

ICUAW is increasingly recognized as major contributor to increased short-term and long-term mortality, prolonged mechanical ventilation, and hospitalization during the acute and subacute phase of the disease and also to poor functional recovery and quality of life thereafter [4, 6, 22, 32]. The current study shows that the maximum decrease of temporal muscle volume, especially, is a strong independent predictor of functional outcome after 3 months. There was also a signal pointing toward an association between increased relative reduction over time and functional outcome, which was not statistically significant. This constellation may be attributable to the longer ICU stay of patients with poor outcome, which comes along with progressive muscle volume loss. However, the association between muscle volume loss and poor functional outcome remained significant after adjusting for the length of ICU stay. Total (persisting) muscle volume appears to play a role for functional outcome as well, as patients with favorable outcome had larger temporal muscle volumes 2 and 3 weeks after the hemorrhage. This is in line with earlier studies showing an association of lower muscle volume and frailty with increased mortality in ICU patients [33, 34].

Controversy exists on whether muscle wasting of ICU patients can be treated. Recent studies indicate that adequate patient nutrition does not prevent the catabolic breakdown of muscle protein but may stimulate muscle protein synthesis during the subacute phase [8, 35, 36]. Early patient mobilization may improve muscle force during and after critical illness [2]. Furthermore, studies on electrical muscle stimulation during the ICU stay yielded promising results [37]. In neurocritical care patients, temporal muscle volume may be a useful biomarker for the monitoring of treatment effects and outcome prognostication.

Some limitations of this study merit consideration. Most importantly, we do not report electrophysiological findings concerning the presence of CIP and CIM, as measurements were not routinely performed in all patients. Furthermore, associations between medication and muscle volume loss were not investigated. Future studies may elucidate the impact of these factors on temporal muscle volume changes. Because of the inclusion criteria, a selection toward poor-grade patients and patients suffering from secondary deterioration occurred. Therefore, changes in temporal muscle volumes of patients with an uncomplicated ICU stay are not well depicted. This may also alter the significance level of differences in muscle volume over time between patients with good outcome or poor outcome, as patients with a favorable clinical course are likely to not receive further imaging in the subacute phase of the disease. This issue can be addressed by future prospective trials predefining time intervals for imaging. A prospective design would also help to clarify the relation between maximum muscle volume loss, length of ICU stay, and poor functional outcome. Because of the retrospective analysis of our data, we cannot draw conclusions on whether more pronounced muscle wasting leads to the need for longer ICU care, vice versa, or both.

## Conclusions

Temporal muscle volume, which is easily assessable on routine head CT scans, progressively decreases during the ICU stay after spontaneous subarachnoid hemorrhage. Because of its association with disease severity and functional outcome, it may serve as biomarker for muscle wasting and outcome prognostication.
